# Genetic diversity among volunteer donors of bone marrow in southeastern Brazil, according to the HLA system

**DOI:** 10.1590/1516-3180.2014.1323619

**Published:** 2014-04-14

**Authors:** Letícia Sarni Roque, Rodolpho Telarolli-Junior, Leonor Castro Monteiro Loffredo

**Affiliations:** I MSc. Biomedical Scientist in the Regional Center for Hematology, Hospital of the Faculdade de Medicina de Ribeirão Preto (FMRP), Universidade de São Paulo (USP), Ribeirão Preto, São Paulo, Brazil; II MD, PhD. Adjunct Professor, Department of Biological Sciences, Universidade Estadual Paulista (Unesp), Araraquara, São Paulo, Brazil; III MD, PhD. Adjunct Professor, Department of Social Dentistry, Universidade Estadual Paulista (Unesp), Araraquara, São Paulo, Brazil

**Keywords:** Histocompatibility antigens, Transplantation, HLA antigens, Bone marrow, Public health, Antígenos de histocompatibilidade, Transplante, Antígenos HLA, Medula óssea, Saúde Pública

## Abstract

**CONTEXT AND OBJECTIVE:**

Checking the histocompatibility of the molecules of the human leukocyte antigen (HLA) system is vital for performing bone marrow transplantation with allogeneic material. The objective of this study was to characterize bone marrow donors according to gender, age, ethnicity and HLA groups at a regional hemotherapy center in Brazil.

**DESIGN AND SETTING:**

Descriptive study on registered donors at a regional hemotherapy center in a public university hospital in the southeastern region of Brazil.

**METHODS:**

The records of 66,780 donors who were registered between 2005 and June 2011 were consulted, and the variables studied were tabulated.

**RESULTS:**

There were equal numbers of male and female donors and 82.8% of them were under 45 years of age. In terms of ethnicity, 77.3% declared themselves to be white, 15.0% mixed race, 5.7% black and 2% others. In terms of immunogenetic characterization, the most frequent HLA-A allelic group was HLA-A*02, with 39.20% of the donors; in the HLA-B allelic group, the most common was HLA-B*35, with 14.18%; while in the HLA-DRB1 allelic group, the most frequent was HLA-DRB1*03, with 17.03%. Comparison between these results and data from the Brazilian Bone Marrow Donor Registry (REDOME) showed that there were demographic and immunogenetic differences due to the history of immigration in the region of Ribeirão Preto, in southeastern Brazil.

**CONCLUSIONS:**

The results reinforce the importance of understanding the demographic and immunogenic profile of regions of Brazil, in order to reduce the waiting time for a histocompatible donor.

## Abstract (pt)

**CONTEXTO E OBJETIVO:**

Para a realização de transplantes de medula óssea com material alogênico, é necessária a verificação de histocompatibilidade das moléculas do sistema HLA (*human leukocyte antigen*), fundamental para o sucesso desses transplantes. O objetivo desta pesquisa foi caracterizar os doadores de medula óssea segundo gênero, idade, etnia e grupos HLA de um centro regional de hemoterapia brasileiro.

**TIPO DE ESTUDO E LOCAL:**

Estudo descritivo dos doadores cadastrados em um centro regional de hemoterapia de um hospital público universitário da região Sudeste do Brasil.

**MÉTODOS:**

Foram consultadas as fichas dos 66.780 doadores cadastrados entre 2005 e junho de 2011 e tabuladas as variáveis estudadas.

**RESULTADOS:**

Encontrou-se distribuição equilibrada entre os gêneros, e 82,8% dos doadores tinham até 45 anos de idade. Quanto à etnia auto-referida, 77,3% se apresentaram como brancos, 15,0% como pardos, 5,7% como negros, os 2% restantes dividindo-se em outras etnias. Quanto à caracterização imunogenética, no grupo alélico HLA-A, o mais frequente foi o HLA-A*02, com 39,20%; no grupo alélico HLA-B, o mais comum foi o HLA-B*35, com 14,18%; no grupo alélico HLA-DRB1, o mais frequente foi o HLA-DRB1*03, com 17,03% do total de doadores. Quando esses resultados são comparados com os dados do cadastro nacional de doadores (REDOME), observam-se diferenças demográficas e imunogenéticas, que se explicam pelo histórico de imigração da região de Ribeirão Preto, no Sudeste brasileiro.

**CONCLUSÕES:**

Os resultados encontrados reforçam a importância de conhecer o perfil demográfico e imunogenético das regiões do Brasil, para reduzir o tempo de espera por um doador histocompatível.

## INTRODUCTION

Organ and tissue transplantation is one of the techniques that have undergone major medical breakthroughs in the last 50 years, thereby modifying the prognoses of millions of patients. In the 21^st^ century, transplantation for therapeutic purposes has become more common because of better understanding of the mechanisms involved in graft rejection and the advent of highly effective immunosuppressive therapies.^1^

In Brazil, the history of transplantation started in the 1960s, shortly after its introduction in the countries that initiated the technique.^2^ The number of transplantations performed in Brazil has been growing year by year, according to the Brazilian Association of Organ Transplants (ABTO). During the 10 years from 2002 to 2011, there was an increase of 350% in the number of transplantations, which totaled 47,062, of which 3.68% were of hematopoietic progenitor cells (bone marrow).^3^

The first bone marrow transplantations were performed in the 1960s, but they became more common in the 1980s, with many applications in cases of diseases such as hematological malignancies or solid tumors, and in other non-hematological disorders such as inborn errors of metabolism, immune deficiencies and other hemoglobinopathies.^4^ ^,^ ^5^ In 2011, 1737 bone marrow transplants were performed, of which 1029 were from autologous donors and 708 from allogeneic donors.^3^

The major histocompatibility complex (MHC) is a set of genes responsible for encoding the histocompatibility molecules in a particular species, which is called the human leukocyte antigen (HLA) in human beings.^6^ The classical histocompatible molecules not only are involved in the immune response as cells presenting peptides to the surface receptors of T lymphocytes, but also are important for understanding mechanisms associated with susceptibility or resistance to certain diseases. These molecules are also strongly associated with allorecognition antigens in organ transplantation, which trigger activation of lymphocytes and the process of graft rejection. Therefore, HLA typing is very important in relation to success in bone marrow transplantation, in which the degree of immunological compatibility between donor and patient is crucial, unlike in most solid organ transplants.^7^ ^-^ ^9^

## OBJECTIVE

The objective of this study was to characterize the bone marrow of volunteer donors at a regional hemotherapy center in a university hospital in the southeastern region of Brazil (a hospital comprising a complex of healthcare institutions serving the region), according to gender, age, ethnicity and HLA groups.

## METHODS

The data source for this study was the records of volunteer bone marrow donors registered at the Regional Center for Hematology, in the teaching hospital of the Ribeirão Preto School of Medicine, University of São Paulo (Hospital das Clínicas, Faculdade de Medicina de Ribeirão Preto, Universidade de São Paulo, HCFMRP/USP), an institution founded in 1990. In addition to Ribeirão Preto, there are hemotherapy centers in several other cities in the state of São Paulo: Araçatuba, Batatais, Bebedouro, Fernandópolis, Franca, Presidente Prudente, Olympia and Serrana. Data from these cities relating to the study subject were also included in this present study. All 66,780 donors registered between January 2005 and December 2011 were included in the study. After the donors had gone through interviews, they were given information about the study and were asked to sign a consent form before making the donation and before their records were updated in the institution's database. Descriptive statistics on variables such as gender, age, self-reported ethnicity and HLA typing were included. The donors were immunogenetically classified by extracting DNA from the complete blood count, using precipitated silica. The polymerase chain reaction-single specific primer (PCR-SSP) was then amplified for each locus using agarose gel and, subsequently, hybridization was performed using specific probes for each locus. The readings were taken from a flow cytometer (Luminex platform) and the results from this platform were interpreted using the HLA Fusion 2.0 software.^6^

## RESULTS

Among the donors, we observed that there was a balance between male and female donors with a slight prevalence of male (50.62%) over female donors (49.37%). Regarding age groups, 83.8% of the donors were of ages up to 45 years (Table 1). In terms of self-reported ethnicity, 77.3% declared themselves to be white, 15.0% mixed race and 5.7% black, and the remaining 2% fell into other categories. 

**Table 1.** Ages of volunteer bone marrow donors registered at the Regional Center for Hematology of the teaching hospital of the Ribeirão Preto School of Medicine, University of São Paulo, 2005 to 2011

Age groups (years)	Donors (%)
18 to 25	23.4
26 to 35	35.5
36 to 45	24.9
46 to 55	14.9
56 and over	1.3

The immunogenetic characterization of the population of bone marrow donors showed a total of 20 different HLA-A alleles for the HLA-A allelic group. HLA-A*02 was the one most frequently present, in 39.20% of the donors, followed by HLA -A*01 in 17.64% (Table 2). In characterizing the HLA-B allelic group, 35 different alleles were found. The most common one was HLA-B*35, accounting for 14.18%, followed by HLA-B*15, with a share of 13.27 % (Table 3). In characterizing the HLA-DRB1 allelic group, 13 different alleles were found, of which HLA-DRB1*03 was the most frequent with a share of 17.03%, followed by HLA-DRB1*04, which was present in 16.58% of the population (Table 4).

**Table 2.** Frequencies of HLA-A alleles among the volunteer bone marrow donors registered at the Regional Center for Hematology of the teaching hospital of the Ribeirão Preto School of Medicine, University of São Paulo, 2005 to 2011

HLA-A alleles	Frequency (%)
A*01	17.64
A*02	39.20
A*03	11.41
A*11	6.04
A*23	5.00
A*24	8.00
A*25	0.84
A*26	2.2
A*29	2.5
A*30	2.8
A*31	1.5
A*32	0.95
A*33	0.72
A*34	0.2
A*36	0.12
A*43	0.01
A*66	0.19
A*68	0.62
A*69	0.01
A*74	0.05

**Table 3.** Frequencies of HLA-B alleles among the volunteer bone marrow donors registered at the Regional Center for Hematology of the teaching hospital of the Ribeirão Preto School of Medicine, University of São Paulo, 2005 to 2011

HLA-B alleles	Frequency (%)
B*07	12.94
B*08	9.28
B*13	2.89
B*14	8.66
B*15	13.27
B*18	7.58
B*27	2.92
B*35	14.18
B*37	1.12
B*38	2.13
B*39	2.98
B*40	3.74
B*41	1.24
B*42	1.30
B*44	7.34
B*45	0.98
B*46	0.04
B*47	0.11
B*48	0.30
B*49	1.35
B*50	1.05
B*51	2.82
B*52	0.42
B*53	0.54
B*54	0.01
B*55	0.20
B*56	0.02
B*57	0.29
B*58	0.14
B*59	0.05
B*67	0.07
B*73	0.00
B*78	0.00
B*81	0.01
B*82	0.03

**Table 4.** Frequencies of HLA-DRB1 alleles among the volunteer bone marrow donors registered at the Regional Center for Hematology of the teaching hospital of the Ribeirão Preto School of Medicine, University of São Paulo, 2005 to 2011

HLA-DRB1 alleles	Frequency (%)
DRB1*01	15.70
DRB1*03	17.03
DRB1*04	16.58
DRB1*07	15.42
DRB1*08	5.54
DRB1*09	1.66
DRB1*10	1.81
DRB1*11	15.59
DRB1*12	1.06
DRB1*13	6.46
DRB1*14	1.32
DRB1*15	1.66
DRB1*16	0.17

## DISCUSSION

To expedite allogeneic transplantation in Brazil, the Brazilian Bone Marrow Donor Registry (REDOME) was created in 1993 and effectively went into operation in 1999 at the National Cancer Institute (INCA).^8^ REDOME is a database in which all bone marrow volunteer donors in Brazil are registered with their personal particulars and HLA typing. From 2005 onwards, the registry began to include registered donors at blood centers and immunogenetic laboratories across the country. This move, coupled with intense social media activities, changed the existing scenario. It led to an exponential growth in the number of registered donors, which totaled 2,307,114 people in June 2011.^10^

The present study shows differences in numbers, in comparison with those found in REDOME, as conducted by Bouzas:^10^ while 77.3% of the donors in Ribeirão Preto were white, 73% in REDOME were white. Regarding the black ethnic group, a disparity was observed in the results, with 12% presented in REDOME and 6% in the present study. For the mixed race group, REDOME presented 10% while the Ribeirão Preto study presented 15%. The explanation for these differences probably lies in the demographic history of the central region of the state of São Paulo, which received a large number of immigrants from European countries in the nineteenth century and, conversely, a smaller flow of slaves of African origin into the region.^11^

In terms of gender, there was a significant disparity between the figures of the Ribeirão Preto study and those of REDOME: while there were equal numbers of male and female donors in the former, female donors accounted for 56% and male donors for 44% in the latter.^10^

Regarding the age group variable, the two databases presented similar compositions, with a predominance of donors between the ages of 18 and 45. This is a very positive sign, since this group of individuals can be considered to be donors for an extended period of time. 

Among the allelic groups found in Ribeirão Preto, the most common of all the groups was HLA-A*02, with a frequency of 39.20%. Among the donors of REDOME, this allelic group was also the most frequent, present in 25.8% of the records.^12^ In the B locus, the allele HLA-B*35 was the most frequent, both in Ribeirão Preto (14.2%) and in REDOME (19.1%). Regarding the DRB1 locus, the one most frequently found in Ribeirão Preto was HLA-DRB1*03 (17.02%), while in REDOME it was HLA-DRB1*04, with 11.90%. The pattern found in Ribeirão Preto was the result, once again, of the demographic peculiarities of the region, due to its history of immigration, thus leading to predominance of alleles of European origin. According to Bone Marrow Donors Worldwide (BMDW), an organization that collects the records of bone marrow donors worldwide, there were 18,513,185 registered donors in 47 countries by the end of 2011.^13^ These donors, of whom 2,307,114 came from Brazil, were dispersed in 21 allelic groups of HLA-A, 35 allelic groups of HLA-B and 13 allelic groups of HLA-DRB1. 

Since Brazil is a multi-racial country, it is essential to increase the number of registered donors, so that this raises the chances that a Brazilian recipient will be given a histocompatible stem cell transplantation.^14^ In late 2011, from preliminary investigations on low or intermediate-resolution tests from the bank of registered donors in REDOME, the probability of finding a locus A, B or DRB1 (6 x 6) compatible donor was 70.53%. In countries that are ethnically more homogeneous, such as Germany and Japan, where the national databases reach a million donors, the possibility of finding a donor for the natives is 85 to 90%.^10^ In bone marrow transplantations, unrelated donors are the option most used today.^15^ However, success in finding a compatible donor depends on race and may be 60 to 70% in the case of Caucasians or only 10 to 20% in the case of other races or mixed race. Using umbilical cord cells is an interesting alternative, but presents the disadvantage of only containing a small number of progenitor cells, which would be insufficient for individuals of greater weight.^16^

Hence, it is important to increase the number of registered bone marrow donors, as well as to better understand the demographics and immunogenetics in different regions of Brazil. In this manner, the waiting time for a histocompatible unrelated donor can be reduced, considering that searching for unrelated donors worldwide takes time, which may often be fatal. Defining the immunogenetic profile of the population of each region of the country is essential for planning strategic campaigns aimed at increasing the number of recipients benefited.^9^ With the objective of catering better for the population's need for bone marrow transplants, future media campaigns aimed at recruiting up donors should take into consideration the characteristics of gender, age, race and the allelic profile of donors already registered in the country's hemotherapy system. It is also important to carry out further studies analyzing associations between the more frequent alleles and self-reported ethnicity.

Creation of REDOME, to which the Regional Center for Hematology of Ribeirão Preto is associated, was a very important step in expediting allogeneic bone marrow transplantation in Brazil. Due to the peculiar characteristics of the population, with a high rate of interracial marriage, it is necessary to have a large number of registered donors in order to find the right donor. Before the creation of this database, it was very common for receivers not to be able to find a compatible unrelated donor in time.

## CONCLUSIONS

There were equal numbers of men and women and predominance of donors under 45 years of age among the donors in this study. More than three quarters of the donors declared themselves to be white and 15% mixed race. In the HLA-A group, HLA-A*02 predominated, accounting for 39.20%, with HLA-A*01 at 17.64%; while in the HLA-B group, HLA -B*35 predominated, accounting for 14.18%, with HLA-B*15 at 13.27%. In the HLA-DRB1 allelic group, HLA-DRB1*03 predominated, accounting for 17.03%, followed by HLA-DRB1*04, present in 16.58% of the population.
